# Giant Aneurysm of Ascending Aorta and Aortic Arch: A Report of a Rare Case

**DOI:** 10.7759/cureus.59393

**Published:** 2024-04-30

**Authors:** Selman Dumani, Ermal Likaj, Laureta Dibra, Alfred Ibrahimi, Arben Baboci

**Affiliations:** 1 Division of Cardiac Surgery, University Hospital Centre “Mother Theresa”, Tirana, ALB; 2 Division of Cardiac Surgery, University Hospital Center "Mother Theresa", Tirana, ALB

**Keywords:** ascending aortic aneurysm, giant aneurysm, circulatory arrest, antegrade cerebral perfusion, total arch replacement

## Abstract

A thoracic aortic aneurysm is considered giant when its diameter exceeds 10 cm. We report a rare case of a giant aneurysm involving the ascending aorta and aortic arch in a 40-year-old man, initially diagnosed as an acute aortic dissection. The patient underwent emergency surgery, during which the ascending aorta and aortic arch were replaced under deep hypothermia and circulatory arrest with selective antegrade cerebral perfusion. Strong teamwork resulted in a favorable postoperative course for the patient.

## Introduction

Aortic aneurysms most commonly involve the aortic root and/or the ascending aorta, accounting for around 60% of the cases, with about 10% affecting the aortic arch. In approximately 10% of cases, more than one portion of the aorta is involved [1]. Surgical treatment remains the gold standard, but managing cases involving the aortic arch remains a significant challenge.

The critical size for complications of an aortic aneurysm is typically 6 cm for the ascending aorta. Once the aorta reaches these dimensions, the likelihood of rupture or dissection is 31% for the ascending aorta [2]. We present a rare case involving both the ascending aorta and the aortic arch, in which the aneurysm dimensions were 13 cm × 10.5 cm. There are few published case reports of giant aneurysms, most of which report on either aneurysms of the ascending aorta or isolated aortic arch aneurysms. Our literature review yielded only one reported case of a giant aneurysm involving both the ascending aorta and the aortic arch [3].

This article was presented previously as a meeting abstract at the 30th Albanian Conference of Surgery and 7th Albanian Congress of Trauma and Emergency Surgery Annual Scientific Meeting held 10-11 of November 2023 as a "Case report and short view".

## Case presentation

**Figure 1:**
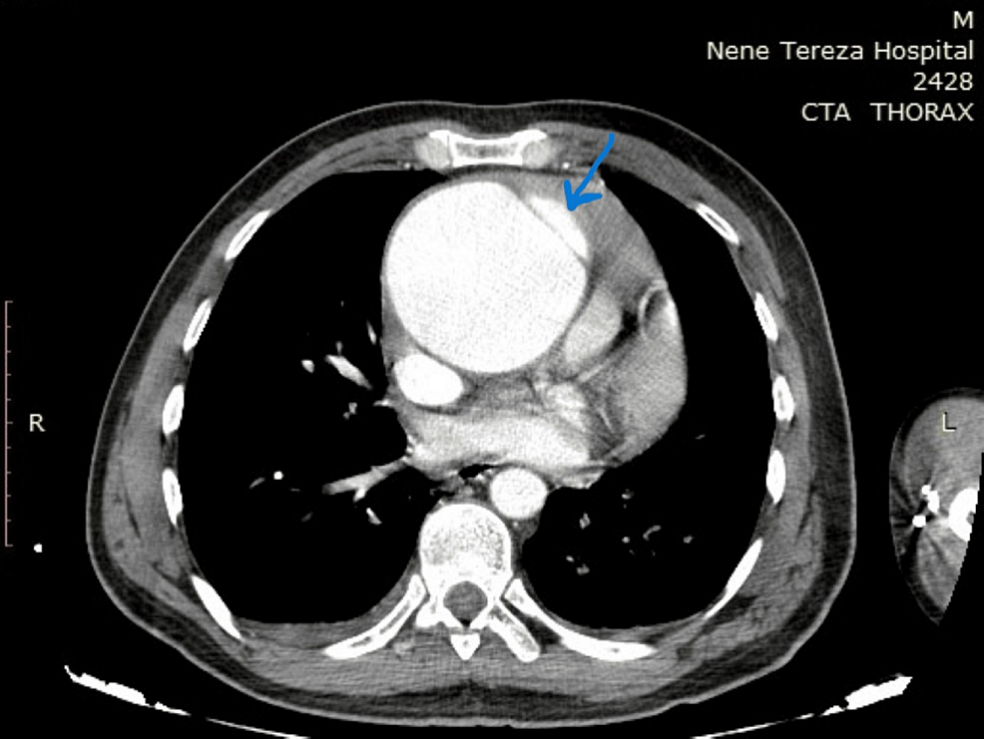
Computed tomography with intravenous contrast The blue arrow indicates the flap dissection.

**Figure 2:**
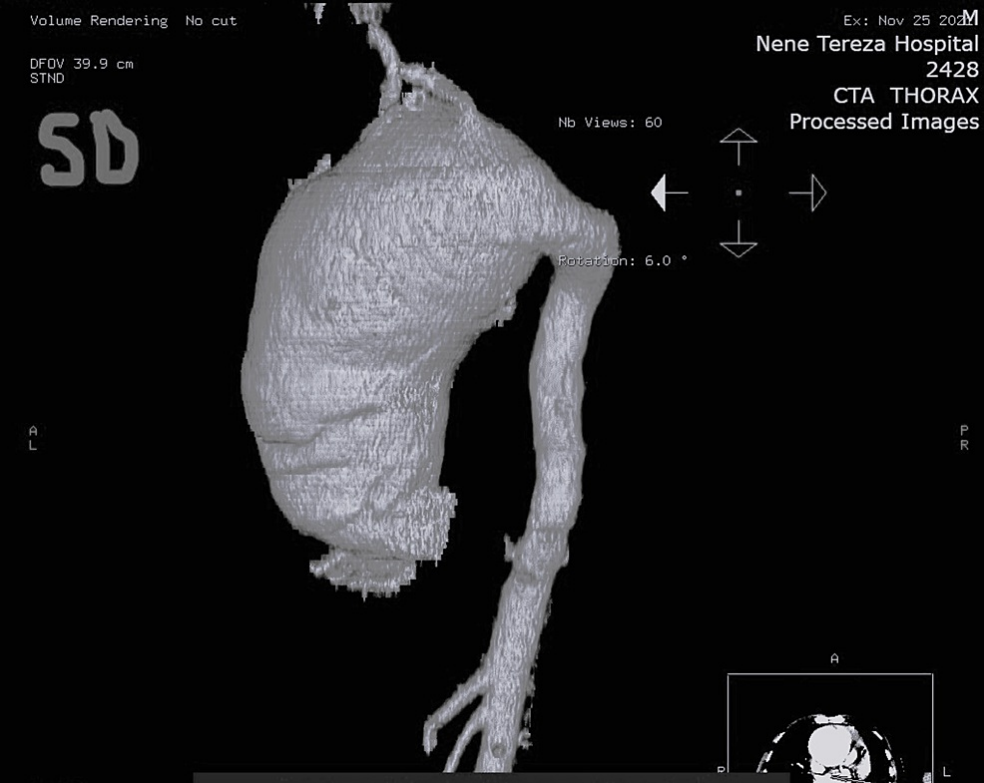
Computed tomography reconstruction of the thoracic aorta

**Figure 3:**
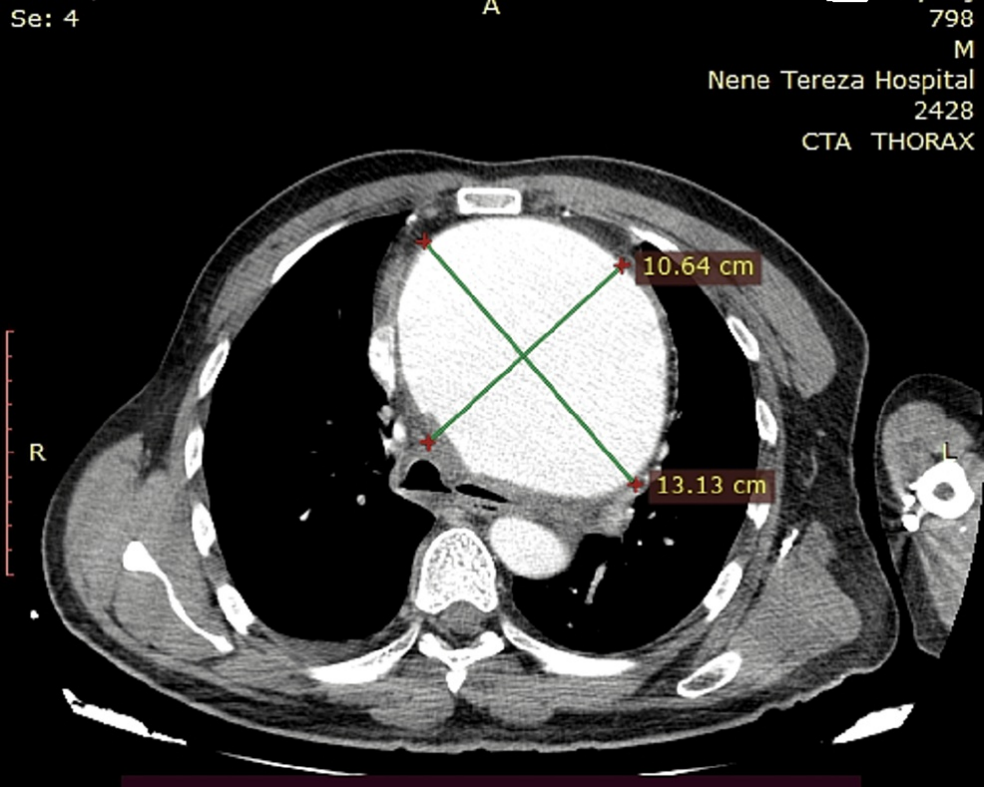
Computed tomography with intravenous contrast The maximal aortic aneurysm dimensions are depicted in the image.

A 40-year-old man presented to the cardiology department with complaints of severe chest pain for about 24 hours, a heart rate of about 95 beats per minute, blood pressure of 160/90 mmHg, and no fever. The cardiology team attempted angiography in the context of ischemic heart disease, but the catheter became stuck in the aortic wall, raising suspicion of aortic dissection. Subsequent computed tomography revealed an acute aortic dissection associated with a giant aneurysm of the ascending aorta and aortic arch (Figures 1-3).

**Figure 4:**
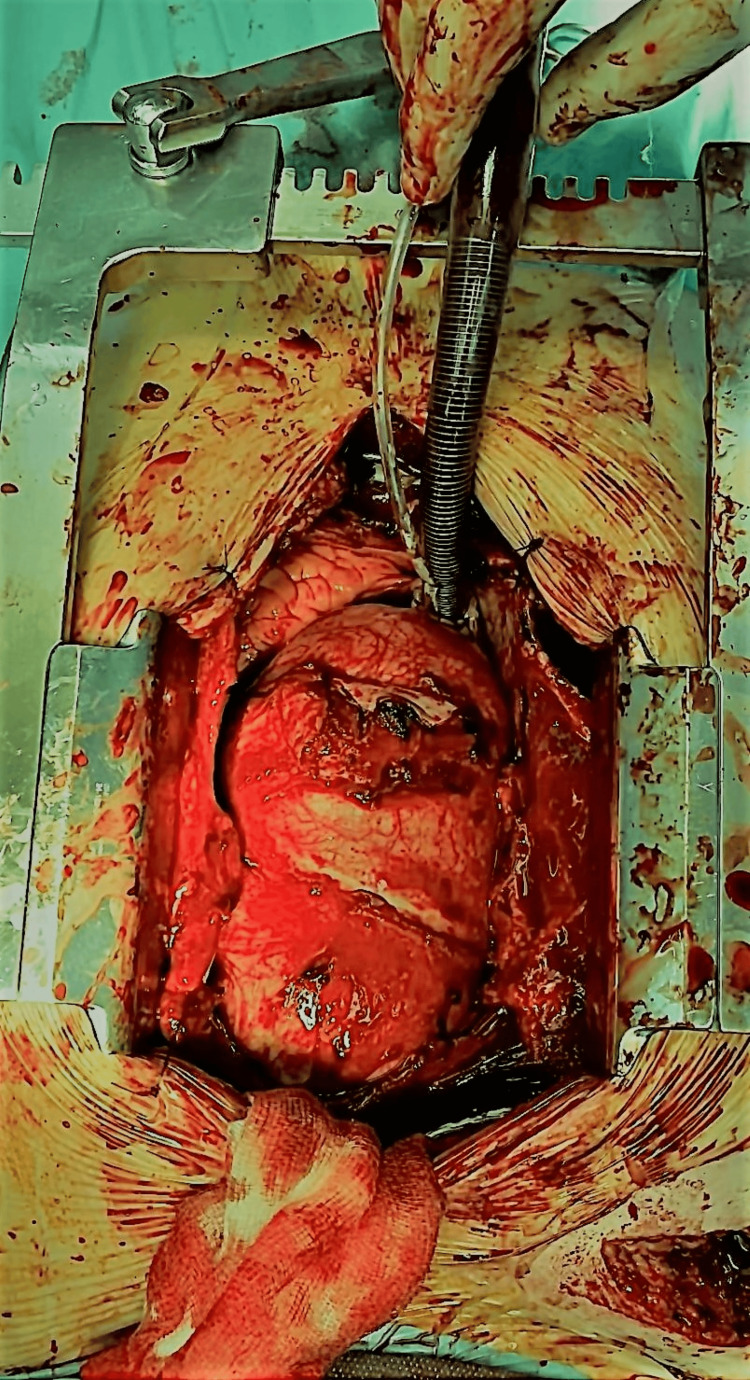
Intra-operative image of the ascending aorta

**Figure 5:**
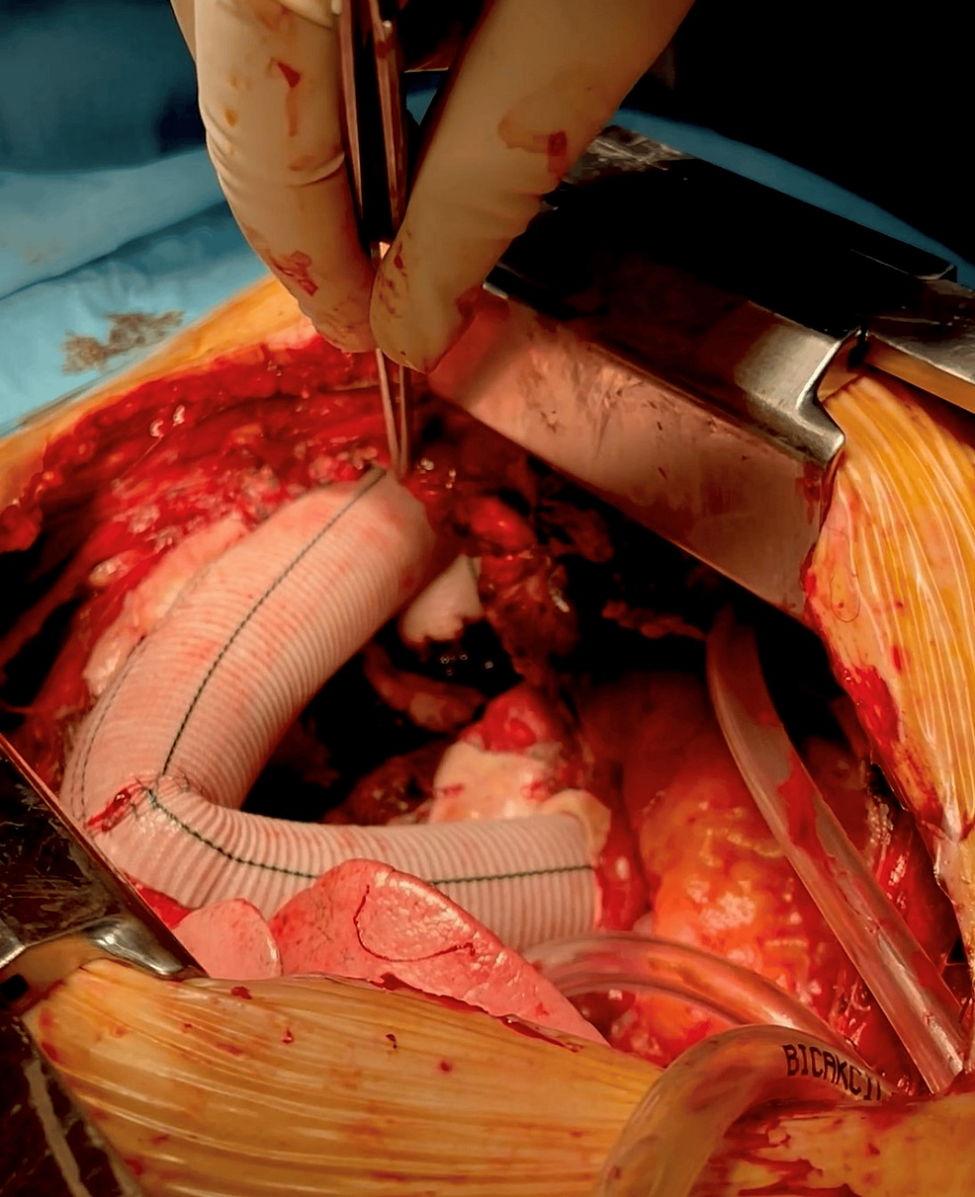
Replacement of ascending aorta and aortic arch with Dacron prosthesis

The patient was referred for emergency surgery. The intervention was carried through a midline incision sternotomy. Right subclavian artery and right atrial appendage cannulation were used to install the heart-lung machine. The ascending aorta and aortic arch were large, making it impossible to clamp the aorta as usual (Figure 4). The patient was cooled down to 25°C. The great vessels were isolated; the distance between them was notably large. The ascending aorta was opened under circulatory arrest with antegrade cerebral perfusion. Crystalloid cardioplegia was delivered through the coronary ostia. A dissected, ulcerated plaque was identified in the aortic wall of the ascending aorta, which likely mimicked a flap dissection. The ascending aorta and aortic arch were resected up to the beginning of the descending aorta, where we performed an anastomosis with a Dacron prosthesis no. 34. Left subclavian, left carotid, and innominate artery buttons were anastomosed to the prosthesis, which was clamped immediately after, and the circulation was restarted. A second Dacron prosthesis no. 34 was used for the replacement of the ascending aorta, and both prostheses were anastomosed. The patient’s chest was closed with gauze and cotton pads due to diffuse bleeding; the gauze and cotton were removed 24 hours later (Figure 5).

## Discussion

The surgical treatment of such giant arch and ascending aorta aneurysms is a rare scenario, posing significant challenges, particularly in emergency conditions. There are only a few cases reported that meet the criteria for giant aneurysms [3-10]. Clinical manifestations can vary from asymptomatic [5] to multiple symptoms, such as airway compression [6-8], acute chest pain [3], cardio-vocal syndrome (Ortner’s syndrome) [4], and superior vena cava syndrome [9,10]. Our patient was referred for an acute aortic dissection based on clinical presentation and computed tomography results. The indication for emergency surgery was unquestionable. 

The intervention plan is critical to the outcomes of every cardiac surgery procedure, particularly in the context of emergency surgery, such as in our case; however, planning for emergency surgery poses a considerable challenge. We implemented meticulous preoperative and operative measures, as we have explained during the case presentation, to optimize the outcome for this patient, as is necessary for major and life-threatening conditions and interventions.

## Conclusions

Surgery remains the gold-standard treatment for such giant aneurysms of the ascending aorta and aortic arch. This surgery involves various procedures, from the way of entry to circulatory arrest, that are different from everyday routine in cardiac surgery. That is why careful management by the entire team is the key to achieving favorable outcomes.
